# New insights into the clinical and molecular spectrum of the *MADD*-related neurodevelopmental disorder

**DOI:** 10.1038/s10038-024-01236-7

**Published:** 2024-03-08

**Authors:** Ghada M. H. Abdel-Salam, Mohamed S. Abdel-Hamid

**Affiliations:** 1 Clinical Genetics Department, Human Genetics and Genome Research Institute, National ResearchCentre, Cairo, Egypt; 2 Medical Molecular Genetics Department, Human Genetics and Genome Research Institute, National Research Centre, Cairo, Egypt

**Keywords:** Disease genetics, Autism spectrum disorders

## Abstract

Biallelic pathogenic variants in *MADD* lead to a very rare neurodevelopmental disorder which is phenotypically pleiotropic grossly ranging from severe neonatal hypotonia, failure to thrive, multiple organ dysfunction, and early lethality to a similar but milder phenotype with better survival. Here, we report 5 patients from 3 unrelated Egyptian families in whom 4 patients showed the severe end of the spectrum displaying neonatal respiratory distress, hypotonia and chronic diarrhea while one patient presented with the mild form displaying moderate intellectual disability and myopathy. In addition, we observed distal arthrogryposis and nonspecific structural brain anomalies in all our patients. Interestingly, cerebellar and brainstem hypoplasia were noted in one patient. Whole exome sequencing identified three novel homozygous variants in the *MADD* gene: two likely pathogenic [c.4321delC p.(Gln1441ArgfsTer46) and c.2620 C > T p.(Arg874Ter)] and one variant of uncertain significance (c.4307 G > A, p.Arg1436Gln). The variants segregated with the disease in all available family members. Our findings confirm that arthrogryposis, genital, cardiac and structural brain anomalies are manifestations of *MADD* which expand the spectrum of *MADD*-related neurodevelopmental disorder. Moreover, they further highlight the convergence of *MADD* variants on different organ systems leading to complex phenotypes.

## Introduction

Initially, the molecular screening of an intellectual disability cohort identified two cases with biallelic pathogenic variants in *MADD* (MAPK -activating death domain-containing protein); these two cases displayed developmental delay and hypotonia [1]. Additional patients harboring *MADD* variants since have been reported [2–5]. One classification divided the phenotype into two groups, the first showing severe developmental delay, hypotonia, hematological anomalies, impairment of the sensory and autonomic nervous system and endo- and exocrine dysfunction and the second with mild to severe developmental delay, hypotonia, speech impairment, and occasionally seizures. The former showed poor prognosis with early lethality [3].

*MADD* gene [MIM#603584] encodes a MAPK-activating protein that is involved in the regulation vesicle trafficking, activity of the Rab3 and Rab27 small GTPases, tumor necrosis factor-alpha (TNF-α) induced signaling and prevention of cell death [6]. The *MADD* pre-mRNA undergoes extensive splicing leading to expression of at least seven different isoforms that are ubiquitously expressed in human tissues [7, 8].

Further clinical descriptions of patients with *MADD*-associated neurodevelopmental disorders can be helpful to understand the variability of the phenotype and confirm the diagnostic criteria. We present here the clinical and molecular description of five new cases from three unrelated families and provide new insights into the phenotypic variability in *MADD*-associated neurodevelopmental disorder along with a review of the literature.

## Patients and methods

The study included 5 patients from 3 unrelated families from Egypt. Patients were referred to Brain Malformations Clinic at National Research Centre (NRC), Cairo for diagnosis and counseling. All patients were subjected to full medical history taking including prenatal, natal and postnatal histories with special emphasis on developmental history, seizure nosology and any other medical conditions. Three generations pedigree construction, complete general examination, full neurological assessment and anthropometric measurements at different ages (supplementary Table 1) were performed. Parents and available family members were also examined. Other investigations including neuroimaging, EEG, ophthalmological evaluation, auditory brain stem response, abdominal ultrasound, echocardiography, thyroid profile, karyotyping and metabolic screening were also pursued.

### Exome sequencing

Genomic DNA of the patients, parents and available family members was extracted using a standard extraction procedure. A signed informed consent was obtained from the parents of each family and our study was approved by the Medical Research Ethics Committee of NRC. Exome sequencing (ES) was performed for one patient from each family (Patients 1, 3 and 5) using SureSelect Human All Exome 50 Mb Kit (Agilent, Santa Clara, CA, USA) and Illumina HiSeq2000 (Illumina, San Diego, CA, USA). Details of ES methods, variant prioritization, and segregation analysis are presented in supplementary Methods.

## Results

### Clinical data

All patients were born at term (40 weeks of gestation) to healthy consanguineous parents of Egyptian ethnicity. Decreased fetal movements were noted during the pregnancies. All had normal acylcarnitine profile, organic acids, karyotype, auditory brain response, ophthalmologic evaluation and abdominal ultrasound. However, they all showed high lactate levels (Table 1).

**Table 1:** Clinical features of our patients with homozygous *MADD* variants compared with the literature

	Family 1		Family 2		Family 3	Total in this study	Schneeberger et al. [3]		Abu-Libdeh et al. [4]
	Patient 1	Patient 2	Patient 3	Patient 4	Patient 5	5 Patients	Group 1 (14)	Group 2 (9)	7 patients
Sex	Female	Male	Male	Female	Male	3 males /2 females	5 males /9 females	7 males /2 females	5 males/ 2 females
**Consanguinity**	+		+		+	3/3 (100%)	4/12 (33%)		
**Decreased fetal movements**	+	+	+	+	+	5/5 (100%)	NA	NA	1/3 (33%)
**Specific facial features and hair color**	High forehead, full cheeks, and blond hair	High forehead and black hair	High forehead, posteriorly rotated ears, and black hair	High forehead and black hair	Wide nasal bridge, low hanging columella, and yellow hair		High forehead, depressed nasal bridge, and small mouth 14/14	High forehead, broad and depressed nasal bridge 9/9	NA
**Developmental delay**	Profound	Profound	Profound	Profound	Moderate (could sit and grasp objects)	4 profound/ 1 moderate	14 severe	5 severe 2 moderate 2 mild	NA
**Age at last examination**	16 months	25 days	6 months	10 months	15 months				
**Anthropometric measures at the age of last examination**									
**OFC in cm (SD)**	41 (−4.3 SD)	35 (−0.5 SD)	42 (−1 SD)	43 (−1.1 SD)	45.5 (−1.7 SD)	1/4 (25%) (<−2 SD)	6/13 (46%) (<−2 SD)	0/7 ( < −2 SD)	1/6 (16%) (<−2 SD)
**Length in cm (SD)**	73 (−2.3 SD)	48 (−1 SD)	62 (−2.6 SD)	64 (−2.3 SD)	75 (−2.3 SD)	4/5 (75%) (<−2 SD)	13/13 (100%) (<−2 SD)	1/7 (14%) (<−2 SD)	4/6 (67%) (<−2 SD)
**Weight in g (SD)**	6,500 (−4 SD)	2,700 (−4.9 SD)	4,900 (−3.7 SD)	6,500 (−2.4 SD)	8,500 (−2.6 SD)	5/5 (100%) (<−2 SD)	10/13 (77%) (<−2 SD)	2/9 (22%) (<−2 SD)	6/7 (86%) (<−2 SD)
**Neurological signs**									
**Axial Hypotonia**	+	+	+	+	+	5/5 (100%)	14/14 (100%)	6/9 (67%)	7/7(100%)
**Peripheral tone/reflexes**	Brisk reflexes	Hyporeflexia	Brisk reflexes	Hyporeflexia	Hyporeflexia	NA	NA	NA	NA
**Seizures**									
**Yes/No**	Yes	No	Yes	Yes	No	3/5 (60%	9/14 (65%)	6/9 (66%	1/7 (14%)
**Type**	GTC	—	GTC	GTC	—	3/3 GTC	3 febrile 2 related to hypoglycemia 1 focal 1 status 2 NA	2 focal/ 2 absence 1 GTC 1 NA	NA
**Onset**	One month	—	9 months	4 months	—	3/3 (1st year of life)	NA	NA	First months of life
**Response to AED**	Controlled	—	Controlled	Controlled	—	3/3 (controlled)	NA	NA	NA
**EEG**	—	—	Normal theta and delta waves	NA	Normal theta and delta waves		9/13 (abnormal)	6/7 (abnormal)	NA
**High CPK**	—	—	NA	+	+	2/4 (50%)	NA	NA	NA
**EMG/nerve conduction**	Normal	NA	—	—	Myopathy	1/4 (25%)	NA	NA	NA
**Brain imaging**									
**Cortical atrophy**	+	NA	+	+	+	4/5 (80%)	5/14 36%) Increased intra- and extra-axial CSF spaces	1/6 (17%) Increased intra- and extra-axial CSF spaces	NA
**Under-opercularization**	+ (Asymmetric)	NA	+	+	+ (Symmetric)	4/4 (100%)	NA	NA	NA
**Hypogenesis corpus callosum**	+	NA	+	+	+	4/4 (100%)	NA	NA	NA
**Ventricular dilatation**	+ (mild)	NA	+ (mild)	+ (mild)	+ (mild)	4/4 (100%)	-	NA	2/7 (28.5%)
**Asymmetry of ventricles**	+	NA	—	—	+	2/4 (50%)	—	NA	NA
**Cerebellar and/or pontine hypoplasia**	+/+	NA	—	—	—	1/4 (25%)	—	NA	1/7 (14%)
**Mega cisterna magna**	+	—	—	—	—	1/4 (25%)	2/14 (14%)	NA	1/7(14%)
**Other Manifestations**									
**Ophthalmologic examination**	Normal	Normal	Normal	Normal	Normal		8/14 (esotropia)	1/9 (esotropia)	NA
**Genitalia**	Normal	undescended testis	Normal	Normal	Normal	1/3 (30%)	4/5(66%)	4/7 (57%)	4/5 (80%)
**Anemia**	+	+	+	+	+ (Mild)	5/5 (100%)	13/14 (93%)	NA	7/7 (100%)
**Thrombocytopenia**	—	+	—	+	Thrombocytosis	2/5 (40%)	5/14 (35%)	NA	1/7 (14%)
**Blood transfusion**	—	+	—	+	—	2/4 (50%)	NA	NA	4/7(57%)
**Arthrogryposis**	**+ (**Bilateral clenched hands, rocker bottom, malposed toes)	**+** (Bilateral clenched hands, severe talipes equinovarus)	+ (Rocker bottom)	+ (Rocker bottom)	+ (Very mild talipes equinovarus)	5/5 (100%)	6/14 (42.8%)	2/9 (22%)	4/7 (57%)
**Metabolic acidosis**	—	**+**	**+**	**+**	—	3/5 (60%)	NA	NA	5/7 (71%)
**Lactate**	High	High	High	High	High	5/5(100%)	NA	NA	4/7 Normal lactate
**Recurrent infections/ diarrhea**	+/−	+/+ Bloody)	**+/+**	**+/+**	−/−	4/5/ (80%) 3/5 (60%)	10/14 (71%)	1/7	7/7(100%)
**Abnormal glucose level**	—	—	+ (Hyperglycemia)	+ (Hyperglycemia)	—	2/5 (40%)	12/13 (92%) (Neonatal hypoglycemia)	0/7	NA
**Congenital heart disease**	—	PDA, PH	-	LVH, PFO	ASD	3/5 (60%)	2/14 (14%)	NA	7/7 (100%)
**Hepatomegaly**	—	—	—	—	—	NA	4/14 (28%)	NA	—
**Alive/dead**	Died at 20 months	Died at 45 days	Died at 2 year	Died at 11 months	Still alive	4/5 (80%)	7/14 (50%)	1/9 (11%)	6/7 (86%)
**Variant**	c.4321delC	c.4321delC	c.4307 G > A	c.4307 G > A	c.2620 C > T	Supplementary Tables 3 and 4			
**Effect on protein**	p.Gln1441Argfs Ter46	p.Gln1441Argfs Ter46	p.Arg1436Gln	p.Arg1436Gln	p.Arg874Ter				

*AED* antiepileptic drugs, *ASD* atrial septal defect, *CPK* creatinine phosphokinase, *EEG* electroencephalogram, *EMG* electromyogram, *GTC* generalized tonic-clonic seizures, *PDA* patent ductus arteriosus, *PH* pulmonary hypertension, *PFO* patent foramen ovale, *LVH* left ventricular hypertrophy

### Family 1 (*Patient 1)*

**Fig. 1:**
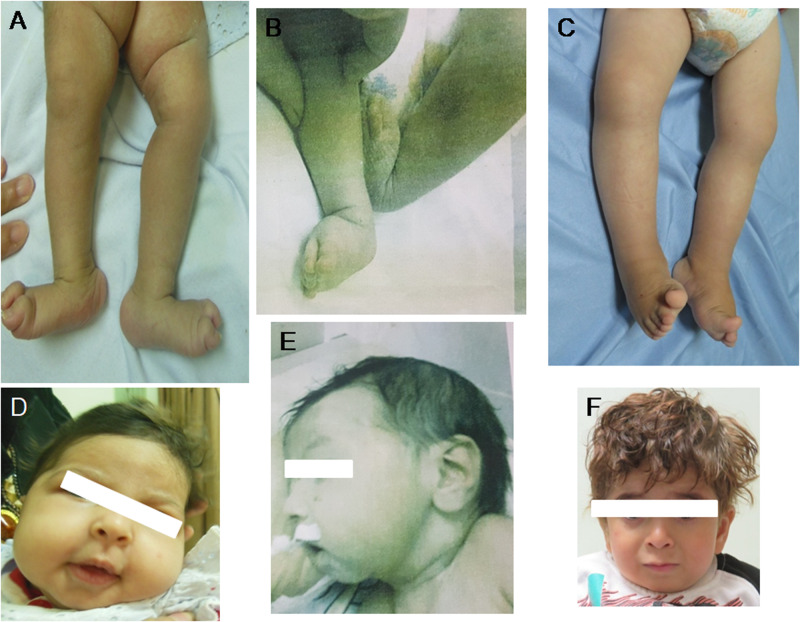
Clinical findings of our patients. Photographs of lower limbs for **A** Patient 1 at the age of 3 months, **B** Patient 2 at the age of 1 month **C** Patient 5 at the age of 15 months showing different severity of arthrogryposis. Frontal facial photographs of **D, E** Patient 1 and Patient 2 at the same ages showing myopathic facial expression with open mouth, high forehead, and full cheeks. The mildly affected **F** Patient 3 had minor facial dysmorphism with wide nasal bridge and low hanging columella

**Fig. 2:**
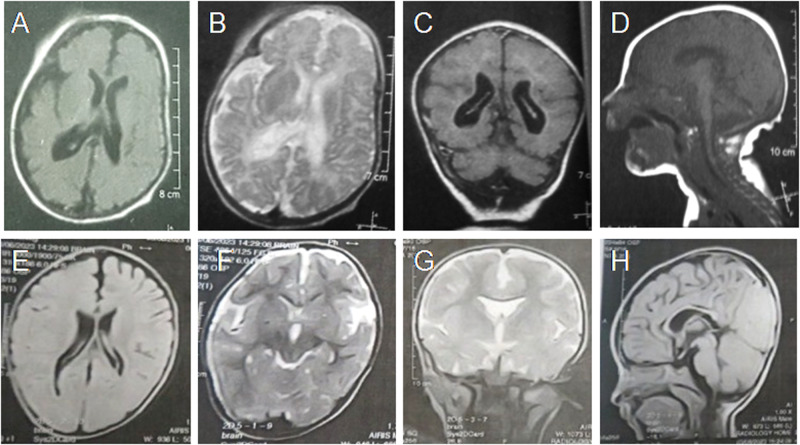
Brain MRI findings of our patients. First row is for Patient 1 at the age of 3 months showing **A, B** mild asymmetric ventriculomegaly, under-opercularization, **C, D** hypogenesis of corpus callosum and cerebellar and brainstem atrophy. Second row is for Patient 5 at the age of 15 months that showed **E, F** asymmetric ventriculomegaly, under-opercularization, **G** cavum septum pellicidum, and **H** hypogenesis of corpus callosum

The female proband was preceded by a healthy sister. Her birth weight was 1800 g (−2.8 SD). Unfortunately other anthropometric results were not recorded. She developed generalized tonic-clonic seizures at the age of one month that showed good response to valproate. On physical examination at the age of 3 months, weight was 4200 g (−2.9 SD), length was 58 cm (−1SD), and occipitofrontal circumference (OFC) was 36.5 cm (−2.3 SD). She appeared irritable displaying excessive crying. She had axial hypotonia and contracture deformities of ankles (Fig. 1) with fully extended knees and clenched hands. In addition, she had dystonic-like movements. Brain MRI at that age showed brain atrophic changes, under-opercularization, ventriculomegaly, hypoplastic corpus callosum, cerebellum and brainstem hypoplasia (Fig. 2). Echocardiography, electromyography and sensory nerve conduction studies showed normal results. She developed pneumonia and died at the age of 20 months.

### Family 1 (*Patient 2)*

He is the youngest brother of Patient 1. His birth weight was 2500 g (−1.8 SD) and birth OFC was 34 cm (−1 SD). At birth, clenched hands and contracture deformities in the ankles were noted (Fig. 1). At the age of 25 days, he was admitted to NICU for 10 days because of cyanosis and the episodes of apnea. He developed bloody diarrhea thereafter. On physical examination at that age, weight was 2700 g (−4.9 SD), length was 48 cm (−1 SD) and OFC was 35 cm (−0.5 SD). Myopathic facies with open mouth, high forehead, long philtrum, and low set ears were noted. The boy had undescended testis. Neurological examination showed static encephalopathy, lethargy with severe hypotonia and hyporeflexia. The only notable abnormal laboratory findings were severe microcytic anemia, thrombocytopenia, and prolonged bleeding profile that nictitated blood transfusion. Brain ultrasound showed normal results. He died suddenly at the age 45 days because of pulmonary hemorrhage.

### Family 2 (*Patient 3)*

He is the first child born to family 2. His birth weight was 2000 g (−2.8 SD). At the age of 2 months, the boy experienced recurrent episodes of vomiting and diarrhea. Generalized seizures began at 9 months of life that showed reasonable control on a combination of valproate and clonazepam. His development was significantly delayed. He did not develop head control or purposeful movement, and he never learned to roll over or sit independently. He had central and peripheral hypotonia with brisk reflexes.On physical examination at the age of 6 months, weight was 4900 g (−3.7 SD), length was 62 cm (−2.6 SD), and OFC was 42 cm (−1 SD). He was noted to have mild talipes equinovarus and rocker bottom feet. Plagiocephaly, nystagmus, and low set simple ears were also noted. He developed frequent chest infections that required recurrent hospital admissions and died because of respiratory failure before two years old.

### Family 2 (*Patient 4)*

She is the youngest sister of Patient 3. Detailed birth records were not available. Failure to thrive and developmental delay were noted in the first 3 months of life. At the age of 4 months, respiratory distress and generalized tonic–clonic seizures were observed. Therefore, the girl was admitted to PICU for 10 days. Her seizures were reasonably controlled with phenytoin. At that age, her axial tone was poor and she was unable to support her head. Her face was myopathic but otherwise not dysmorphic. She had high forehead and her ears were slightly simply formed. Mild talipes equinovarus was also noted. Echocardiography revealed left ventricular hypertrophy and patent foramen ovale. She was admitted again to PICU at the age of 5 months because of status epilepticus and metabolic acidosis. Her condition deteriorated and developed respiratory failure therefore mechanically ventilated until death at the age of 11 months. At the age of 10 months, her weight was 6500 g (−2.4 SD), length was 64 cm (−2.3 SD) and OFC was 43 cm (−1.1 SD). Laboratory blood investigations showed high CPK, lactate, and pancytopenia. She had a history of blood transfusion. Episodes of hyperglycemia were documented. Brain MRI showed mild atrophic changes in the form of prominent extra-axial CSF spaces and dilated ventricular system.

### Family 3 (*Patient 5)*

He was the first child born to consanguineous parents. The previous pregnancy ended with spontaneous first trimester miscarriage. His birth weight was 2800 g (−1 SD). He exhibited clubfeet which had been corrected repeatedly by splints with fair improvement. Echocardiographic evaluation at the age of 3 months documented atrial septal defect that later closed spontaneously. He first presented to our clinic when he was 8-months old because of global developmental delay. On physical examination, impaired gross motor development and limitation in the movements of distal joints (difficulty in pinch and grasp functions) were evident. His weight was 7000 g (−1.8 SD), length 68 cm (−1.8 SD), and OFC 42 cm (−1.8 SD). Initial development was delayed (partial neck control was possible at the age of 8 months and he was able to sit independently at the age of 18 months). He did not have any speech or sounds to communicate. He could follow objects, attend to his surroundings, and follow simple commands. There was no history of epilepsy. Hypotonia and hyporeflexia were documented. Brain MRI showed asymmetric ventriculomegaly, under-opercularization, cavum septum pellucidum, and hypogenesis of corpus callosum (Fig. 2). Electromyography and sensory nerve conduction studies showed myopathy. Laboratory blood investigations showed normal glucose level and CPK but mild microcytic anemia.

### Molecular findings

**Fig. 3:**
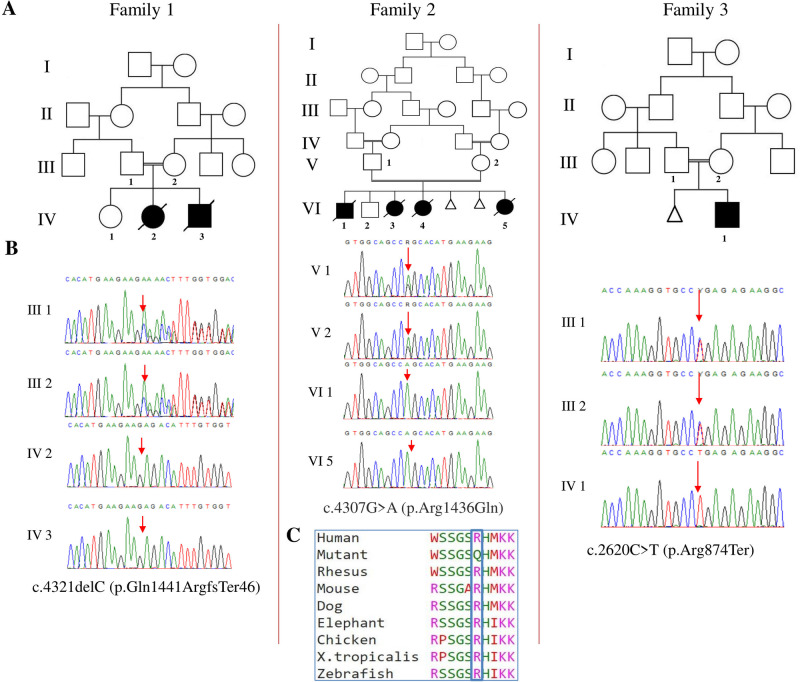
Pedigrees and genetic findings of our families. **A** Pedigrees of the three families. **B** Portions of the sequencing electropherograms showing the new *MADD* variants identified in our study. Arrows indicate site of variants. **C** Schematic diagram showing the conservation of the p.Arg1436 residue across different species

Exome sequencing revealed three new variants in *MADD* in our three families. A frameshift variant, c.4321delC (p.Gln1441ArgfsTer46) in exon 30 of the gene (NM_003682.3) was detected in Family 1 and a missense variant in the same exon (c.4307 G > A, p.Arg1436Gln) was identified in Family 2. On the other hand, a nonsense variant c.2620 C > T (p.Arg874Ter) in exon 15 was detected in Family 3. Variants were homozygous in the patients and confirmed by Sanger sequencing to be heterozygous in their respective parents and healthy siblings (Fig. 3). The missense variant p.Arg1436Gln was predicted to have a damaging effect on the protein function by different bioinformatic tools (Supplementary Table 2**)**. It is classified as variant of uncertain significance according to ACMG classification of variants while the remaining two variants are classified as “likely pathogenic”. Details of the in-silico prediction of our new variants, allele frequency in gnomAD, and ACMG classification are depicted in Supplementary Table 2.

## Discussion

We describe the clinical manifestations observed in 5 patients with homozygous novel likely disease-causing variants in the *MADD* gene that presented with neurodevelopmental delay as well as arthrogryposis. Considering the previously reported patients, a variable neurodevelopmental delay was observed ranging from remarkable global developmental delay with early lethality to mild intellectual disability and longer survival [3, 4]. Four of our patients presented with the severe end of the spectrum while one showed the milder presentation. Hypotonia is a cardinal feature of *MADD* gene dysfunction being reported previously in 100% of cases in the lethal group [3, 4]. Likewise, failure to thrive (>70% of cases), hematological abnormalities (>90% of cases), and neonatal hypoglycemia (>90% of cases) are consistent features in the lethal group [3, 4]. Microcephaly (<−2 SD) was documented in 26% of the previously reported patients [3, 4] and 14% of our patients (one case). In contrast, the initial report showed relative macrocephaly in one patient [2]. Based on our results and those reported before [3, 4], the deceleration in the weight and length are more significant compared to the head circumference, thus giving the impression of relative macrocephaly. Endocrine dysfunction was reported in 80% of patients of the lethal group [3] but none of our patients. Given the early lethality, it is possible that some phenotypic traits are underreported in the existing literature. Frequencies of clinical features reported to date among patients with *MADD* variants compared with our cohort are provided in Table 1.

Arthrogryposis appears likely to represent an important feature of the condition. Notably, it was observed in all our patients (100%) and in 41% of those reported previously although provided no further details [3, 4]. It is typically congenital and shows a distal pattern of hands and feet although contracture of knee and elbow was observed in 2 patients (2/14; 14%) in a previous report [3]. Taken together with our present findings, these data provide compelling evidence for the association of arthrogryposis with *MADD* deficiency. Further reports would help to substantiate this observation. Therefore, we suggest that arthrogryposis should be added as one of the characteristic features for *MADD-*related neurodevelopmental disorder.

Patient 5 showed mild intellectual disability and myopathy whereas Patient 4 had a high CPK level. In contrast, Patient 1 in our cohort showed normal electromyogram (EMG) and nerve conduction studies. The defective neuromuscular transmission was seen in Madd-deficient mice [6]. However, the interaction of MADD with KIF1A that is a motor protein mediating the axonal transport of Rad3-positive vesicles [9] and the reduced pain sensations among patients of group 1 of Schneeberger and co-authors [3] gave evidences of hereditary sensory and autonomic neuropathy type II features among patients of this group. These results seem challenging. We recommend EMG and nerve conduction studies as well as the CPK measurement in any case with *MADD* deficiency. Although heterozygous missense variants in *MADD* have been also associated with muscular dystrophy [10], but the heterozygous parents (of our Patients 3 and 4 who had missense variant) were unaffected.

Brain MRI showed normal results in most of the reported cases (19/27; 70%). However, increased intra-and extra-axial CSF spaces were reported in 8 patients (8/27; 30%) and two also showed mega cisterna magna [3, 4]. Moreover, a hypoplastic pituitary was found previously in 2 patients; (2/27; ~7%) and was considered one of the main risk factors of hormone deficiencies [3]. The increased intra- and extra-axial CSF spaces are either indicative of brain atrophy or an increased production of cerebrospinal fluid in these patients. The cerebrospinal fluid has an important dynamic role in the central nervous development system [11]. In this study, we also noted asymmetric dilatation of lateral ventricles, hypoplastic corpus callosum and under-opercularization in four patients. These non-specific features argue against their clinical utility. Unexpectedly, Patient 1 in this study had brainstem and cerebellar hypoplasia. Reports of additional cases are needed to confirm the association between these brain imaging findings and *MADD* dysfunction.

Genital anomalies (micropenis and undescended testis) have been previously reported in 70% (12/17) of male patients with *MADD* variants and also in one of our male patients (33%). Thus, they appear likely to represent an important feature of the condition [3, 4].

Interestingly, congenital heart disease was not described as a consistent feature, reported in 2/14 (14%) of cases [3]. The cases we describe and those described by Abu-Libdeh et al. [4], have a much more prevalence of congenital heart disease showing 80% and 100%, respectively. It may be related to the periodic apnea described in these patients as 30% of cases showed variability in heart rate [3] but the pathogenesis of the periodic apnea is still unclear. Therefore, we propose that regular cardiac monitoring is performed.

Generalized tonic clonic seizures occurred in more than half of our patients and those reported in the literature [3, 4] and typically developed in the neonatal period and showed good response to antiepileptic drugs. The febrile illness and/or hypoglycemia-triggered seizures (usually presenting in the first year of life) are evident in 35% (5/14) of reported patients [3].

In total, 25 different *MADD* variants have been reported in 34 individuals [1–4]. They include 11 missense, 6 nonsense, 3 frameshfit, 4 splice, and one large deletion (encompassing exons 11 to 24). Variants were scattered across the coding region with no hot-spot exons. In this study, we identified three new *MADD* variants including two protein truncating [c.2620 C > T (p.Arg874 Ter) and c.4321delC (p.Gln1441ArgfsTer46)] and one missense [c.4307 G > A (p.Arg1436Gln)]. The two protein truncating variants are predicted to result in nonsense-mediated mRNA decay. On the other hand, the missense variant p.Arg1436Gln affects a highly conserved amino acid residue and was deemed deleterious by multiple bioinformatic tools. In addition, it was not found in our in-house database of >1500 exomes of Egyptian origin. Identification of more patients with this variant and future functional studies are warranted. This would help to confirm its pathogenicity. As such, we raise the total number of *MADD* disease-causing variants to 28 (Supplementary Table 4).

Among the reported variants, only four were recurrent including the p.Arg327* which was reported in 5 unrelated families, in the homozygous form in one family [3] and in the heterozygous state along with other variants in 4 families [1, 3]. Interestingly, all patients carrying the p.Arg327* were of European origin (Supplementary Table 3). The p.Arg327* is present in the heterozygous state in 47 normal individuals (MAF = 0.000029) according to gnomAD version 4, 45 of them were Europeans. Therefore, it is common and may be a founder *MADD* variant among Europeans. Another founder variant (c.2816+1 G > A) was described in 7 patients from 4 unrelated Arab-Muslim families [4]. Additionally, the p.Pro354Leu and p.Ser1213* variants were reported in the heterozygous state twice in two unrelated Europeans families each [1, 3]. Other variants are unique and appear to be private as each found in one family.

Our results support the findings of Schneeberger and co-authors [3] and confirm the absence of phenotype-genotype correlations in patients with *MADD* variants. Missense variants were identified among many patients of the severe end [3]. Likewise, Patients 3 and 4 in our study harboring the missense variant c.4307 G > A (p.Arg1436Gln) presented with a severe neurological phenotype, multiple organ dysfunction and showed early lethality. In comparison, Patient 5 whom carried a homozygous nonsense variant in exon 15 displayed a relatively milder phenotype and longer survival.

In conclusion, our report confirms the core phenotype associated with biallelic *MADD* variants. Additionally, our results suggest that patients with *MADD* variants are likely to have variable degrees of arthrogryposis, structural brain, congenital heart disease, and genital anomalies. The variability of the clinical phenotype of MADD poses challenges to genetic counseling and warrants a comprehensive clinical evaluation and long-term multidisciplinary follow-up of these individuals. Future studies will decipher the precise mechanisms through which *MADD* deficiency leads to arthrogryposis, genital anomalies and structural brain anomalies.

### Supplementary information

Supplementary Tables

Supplementary Methods

## Data Availability

The data that support the findings of this study are available from the corresponding authors upon reasonable request.
