# Supplementary material for: New insights into the clinical and molecular spectrum of the MADD-related neurodevelopmental disorder
Source: J Hum Genet. 2024 Mar 8;69(6):263–70. doi: 10.1038/s10038-024-01236-7 (PMC11126384; doi:10.1038/s10038-024-01236-7)
Supplement: Supplementary file 1 — Supplementary Tables [file 10038_2024_1236_MOESM1_ESM.doc]

**Supplementary Table 1: The anthropometric measures of the 5 patients with MADD variants at different ages.**

|  | **Age** | **Weight in g (SD)** | **Length in cm (SD)** | **OFC in cm (SD)** |
| --- | --- | --- | --- | --- |
| **Patient 1** | 3 months | 4,200 (-2.9 | 58 (-1) | 36.5 (-2.3) |
|  | 16 months | 6,500 (-4.1) | 73 (-2.3) | 41(-4.6) |
| **Patient 2** | at birth | 2,500 (-1.8) | NA | 34 (-1) |
|  | 25 days | 2,700 (-4.9) | 48 (-1) | 35 (-0.5) |
| **Patient 3** | 6 months | 4,900 (-3.7) | 62 (-2.6) | 42 (-1) |
|  | 8 months | 4,500 (-4.3) | 62 (-4.1) | 42.5 (-1) |
| **Patient 4** | 10 months | 6,500 (-2.4) | 64 (-2.3) | 43 (-1.1) |
| **Patient 5** | 7 months | 7,000 (-1.8) | 68 (-1.8) | 42 (-1.8) |
|  | 15 months | 8,500 (-2.8) | 75 (-1.5) | 45.5 (-1) |

**Supplementary Table 2**: In silico analysis and variant classification of our three *MADD* variants

| **Variant (nucleotide change/amino acid change)** | **REVEL^A^** | **CADD^B^** | **SIFT^C^** | **MT^D^** | **PolyPhen^E^** | **PROVEAN ^F^** | **gnomAD allele frequency ^G^** | **ACMG criteria** |
| --- | --- | --- | --- | --- | --- | --- | --- | --- |
| c.2620C>T (p.Arg874Ter) | - | 38 | - | Disease Causing automatic (1) | - | - | 0.00001113 | Likely pathogenic  PM2 moderate  PP3 moderate  PM6 moderate  PP2 supporting |
| c.4307G>A (p.Arg1436Gln) | Uncertain (0.507) | 32 | Deleterious (0) | Disease Causing (1) | Probably damaging (0.992) | Damaging  (-3.63) | 0.00006568 | VUS  PM2 moderate  PM6 moderate |
| c.4321delC (p.Gln1441ArgfsTer46) | - | 38 | - | Disease Causing automatic (1) | - | - | Not present | Likely pathogenic  PM2 moderate  PP3 moderate  PM6 moderate  PP2 supporting |

ACMG: American College of Medical Genetics, VUS: variant of uncertain significance

^A^ REVEL is based on a combination of scores from 13 individual tools: MutPred, FATHMM v2.3, VEST 3.0, PolyPhen-2, SIFT, PROVEAN, MutationAssessor, MutationTaster, LRT, GERP++, SiPhy, phyloP, and phastCons. It ranges from 0 to 1, with higher scores reflecting greater likelihood that the variant is disease-causing.

^B^ CADD predicts a continuous phred-like score that ranges from 1 to 99, higher values indicating more deleterious cases.

^C^ SIFT score (SIFTori). Scores range from 0 to 1. The smaller the score the more likely the SNP has damaging effect.

^D^ MutationTaster score ranges from 0.0 (polymorphism) to 215 (disease causing).

^E^The PolyPhen-2 score ranges from 0.0 (tolerated) to 1.0 (deleterious). Variants with scores of 0.0 are predicted to be benign. Values closer to 1.0 are more confidently predicted to be deleterious.

^F^ PROVEAN: Scores range from -14 to 14. The smaller the score the more likely the SNV has damaging effect.

^G^Allele Frequency data from gnomAD: genome data from gnomAD v4.0.0.

**Supplementary Table 3**: Overview of all reported patients with *MADD* variants with their genotype and ethnicity

| **Ref** | **No of families** | **No of patients** | | **Origin** | **Variant** | **Effect on Protein** |
| --- | --- | --- | --- | --- | --- | --- |
| **Anazi et al. (2017)** | 2 | 2 | 1 | Saudi | c.2930T>G/c.2930T>G | p.Val977Gly/ p.Val977Gly |
|  |  |  | 1 | American | c.593G>A/c.979C>T | p.Arg198His / p.Arg327* |
| **Hu et al. (2019)** | 1 | 2 | 2 | Persian | c.1061C>T/ c.3559del | p.Pro354Leu/ p.Met1187Ter |
| **Schneeberger et al. (2020)** | 17 | 23 | 2 | Caucasian | c.914G>T/ c.(1862+1_ 1863-1)_(3759+ 1_3760-1)del | p.Gly305Val/ p.? |
|  |  |  | 2 | Arabic | c.963+1G>A/ c.963+1G>A | p.? |
|  |  |  | 1 | Caucasian/ native American | c.979C>T/ c.1705+1G>C | p.Arg327*/ p.? |
|  |  |  | 1 | Arabic | c.3119T>G/ c.3119T>G | p.Leu1040Arg / p.Leu1040Arg |
|  |  |  | 1 | South American | c.646C>T/ c.646C>T | p.Arg216*/ p.Arg216* |
|  |  |  | 1 | Caucasian | c.979C>T/ c.979C>T | p.Arg327*/ p.Arg327* |
|  |  |  | 1 | Caucasian | c.4293G>A/ c.4293G>A | p.Trp1431*/ p.Trp1431* |
|  |  |  | 1 | Caucasian | c.979C>T/ c.3760-2A>C | p.Arg327*/ p.? |
|  |  |  | 1 | Arab-Berbers | c.770C>T/ c.770C>T | p. Ser257Phe / p.Ser257Phe |
|  |  |  | 1 | Caucasian | c.979C>T/c.4398delG | p.Arg327*/ p.Leu1467Cysfs*20 |
|  |  |  | 2 | Pakistani/Indian | c.1115C>T/ c.4080delG | p.Pro372Leu / p.Leu1361Serfs*24 |
|  |  |  | 1 | Caucasian | c.1037T>C/ c.1037T>C | p.Leu346Pro / p.Leu346Pro |
|  |  |  | 2 | Caucasian | c.3533_3534 delCT/ c.3848A>C | p.Ser1178Cysfs*18 / p.Tyr1283Ser |
|  |  |  | 2 | Caucasian | c.1061C>T / c.3637_3638 delAG | p.Pro354Leu / p.Ser1213* |
|  |  |  | 1 | Fars | c.3952T>C/ c.3952T>C | p.Trp1318Arg / p.Trp1318Arg |
|  |  |  | 2 | Persian | c.2834T>C/ c.2834T>C | p.Leu945Pro / p.Leu945Pro |
|  |  |  | 1 | Caucasian | c.3637_3638 delAG/ c.4594C>T | p.Ser1213*/ p.Arg1532* |
| **Abu-Libdehet. (2021)** | 4 | 7 | 7 | Arab Muslim | c.2816+1G>A/c.2816+1G>A | p.? |
| **This study** | 3 | 5 | 2 | Egyptian | c.4321delC/ c.4321delC | p.Gln1441ArgfsTer46/ p.Gln1441ArgfsTer46 |
|  |  |  | 2 | Egyptian | c.4307G>A/ c.4307G>A | p.Arg1436Gln / p.Arg1436Gln |
|  |  |  | 1 | Egyptian | c.2620C>T/ c.2620C>T | p.Arg874Ter/ p.Arg874Ter |

**Supplementary Table 4:** The location, type and frequency of allvariants identified so far in the literature in patients with *MADD-*related neurodevelopmental disorders

| **Serial** | **Variant** | **Protein** | **Type** | **Location** | **References** |
| --- | --- | --- | --- | --- | --- |
| 1 | c.593G>A | p.Arg198His | Missense | Exon 3 | **Anazi et al. (2017)** |
| 2 | c.646C>T | p.Arg216* | Nonsense | Exon 3 | **Schneeberger et al. (2020)** |
| 3 | c.770C>T | p.Ser257Phe | Missense | Exon 4 | **Schneeberger et al. (2020)** |
| 4 | c.914G>T | p.Gly305Val | Missense | Exon 4 | **Schneeberger et al. (2020)** |
| 5 | 963+1G>A | Alter splicing | Splice | Intron 4/ Exon 5 | **Schneeberger et al. (2020)** |
| 6 | c.979C>T | p.Arg327* | Nonsense | Exon 5 | **Anazi et al. (2017)**  **Schneeberger et al. (2020)** |
| 7 | c.1037T>C | p.Leu346Pro | Missense | Exon 5 | **Schneeberger et al. (2020)** |
| 8 | c.1061C>T | p.Pro354Leu | Missense | Exon 5 | **Hu et al. (2019)**  **Schneeberger et al. (2020)** |
| 9 | c.1115C>T | p.Pro372Leu | Missense | Exon 6 | **Schneeberger et al. (2020)** |
| 10 | c.1705+1G>C | Alter splicing | Splice | Intron 9/ Exon 10 | **Schneeberger et al. (2020)** |
| 11 | c.(1862+1_ 1863-1)_(3759+ 1_3760-1)del | p.? | Large intragenic deletion | Exons 11-24 | **Schneeberger et al. (2020)** |
| 12 | **c.2620C>T** | **p.Arg874Ter** | **Nonsense** | **Exon 15** | **This study** |
| 13 | c.2816+1G>A | Alter splicing | Splice | Exon 17/Intron 17 | **Abu-Libdehet. (2021)** |
| 14 | c.2834T>C | p.Leu945Pro | Missense | Exon 18 | **Schneeberger et al. (2020)** |
| 15 | c.2930T>G | p.Val977Gly | Missense | Exon 18 | **Anazi et al. (2017)** |
| 16 | c.3119T>G | p.Leu1040Arg | Missense | Exon 19 | **Schneeberger et al. (2020)** |
| 17 | c.3533_3534 delCT | p.Ser1178Cysfs*18 | Frameshift | Exon 23 | **Schneeberger et al. (2020)** |
| 18 | c.3559del | p.Met1187Ter | Nonsense | Exon 23 | **Hu et al. (2019)** |
| 19 | c.3637_3638 delAG | p.Ser1213* | Nonsense | Exon 24 | **Schneeberger et al. (2020)** |
| 20 | c.3760-2A>C | Alter splicing | Splice | Intron 24/Exon 25 | **Schneeberger et al. (2020)** |
| 21 | c.3848A>C | p.Tyr1283Ser | Missense | Exon 25 | **Schneeberger et al. (2020)** |
| 22 | c.3952T>C | p.Trp1318Arg | Missense | Exon 27 | **Schneeberger et al. (2020)** |
| 23 | cc.4080delG | p.Leu1361Serfs*24 | Frameshift | Exon 28 | **Schneeberger et al. (2020)** |
| 24 | c.4293G>A | p.Trp1431* | Nonsense | Exon 30 | **Schneeberger et al. (2020)** |
| 25 | **c.4307G>A** | **p.Arg1436Gln** | **Missense** | **Exon 30** | **This study** |
| 26 | **c.4321delC** | **p.Gln1441ArgfsTer46** | **Frameshift** | **Exon 30** | **This study** |
| 27 | c.4398delG | p.Leu1467Cysfs*20 | Frameshift | Exon 31 | **Schneeberger et al. (2020)** |
| 28 | c.4594C>T | p.Arg1532* | Nonsense | Exon 32 | **Schneeberger et al. (2020)** |

Novel variants identified in this study are in bold.
