# Supplementary material for: New insights into the clinical and molecular spectrum of the MADD-related neurodevelopmental disorder
Source: J Hum Genet. 2024 Mar 8;69(6):263–70. doi: 10.1038/s10038-024-01236-7 (PMC11126384; doi:10.1038/s10038-024-01236-7)
Supplement: Supplementary file 2 — Supplementary Methods [file 10038_2024_1236_MOESM2_ESM.doc]

**Supplementary Methods**

***Whole Exome Sequencing***

Whole exome sequencing was performed using SureSelect Human All Exome 50 Mb Kit (Agilent, Santa Clara, CA, USA) and Illumina HiSeq2000 (Illumina, San Diego, CA, USA). The obtained sequences were aligned to UCSC human genome GRCh37/hg19 and variants were verified through the GATK pipeline. Annotation of variants was done using BaseSpace Variant Interpreter Server. Identified variants were checked against public genetic databases like Genome Aggregation Database (gnomAD, https://gnomad.broadinstitute.org/), 1000 Genomes (www.1000genomes.org), dbSNP (<http://www.ncbi.nlm.nih.gov/SNP/>) and our in-house database of more than 1500 exomes. Pathogenicity of the detected variants was predicted using SIFT (https://provean.jcvi.org/protein), PolyPhen-2 (https://genetics.bwh.harvard.edu/pph2/), MutationTaster (<https://www.mutationtaster.org/)>, CADD (<https://cadd.gs.washington.edu/>), and REVEL (<https://genome.ucsc.edu/cgi-bin/hgTrackUi?db=hg19&g=revel>).

***Segregation analysis using Sanger sequencing***

Segregation analysis of the identified variants in the parents and available family members was conducted by PCR amplification of exons 15 and 30 using specific primers designed by Primer3 software. Our standard PCR cycling conditions were: initial denaturation at 95°C for 10 min; 30 cycles of denaturation at 95°C for 1min; annealing at 62.5°C for 1min; extension at 72°C for 1min, and a final extension at 72°C for 10 min. PCR products were purified using Exo-SAP PCR Clean-up kit (Fermentas, Germany) and sequenced in both directions using the BigDye Terminator v3.1 Cycle Sequencing Kit (Applied Biosystems, Foster City, CA, USA) and analyzed on the ABI Prism 3500 Genetic Analyzer (Applied Biosystems) according to manufacturer's instructions.
